# Retraction Note: Atomic insight into hydration shells around facetted nanoparticles

**DOI:** 10.1038/s41467-024-50636-y

**Published:** 2024-07-25

**Authors:** Sabrina L. J. Thomä, Sebastian W. Krauss, Mirco Eckardt, Phil Chater, Mirijam Zobel

**Affiliations:** 1 Department of Chemistry, University of Bayreuth, Universitätsstr.30, 95440 Bayreuth, Germany; 2 Diamond Light Source, Harwell Science & Innovation Campus, Didcot, Oxfordshire OX11 0DE UK

Retraction to: *Nature Communications* 10.1038/s41467-019-09007-1, published online 01 March 2019

The authors have retracted this Article after they discovered during subsequent work^1^ that the interpretation of the observed dd-PDF signals, which were assumed to originate from the hydration shell, is incorrect. Rather, the highly sensitive dd-PDF signal stems from short-range order signal of ethanol-water motifs which were detected alongside iron oxide signals in the aqueous dispersion. The ethanol traces within the dispersions are a remnant of the purification steps following particle synthesis. Since new experimental evidence and related literature^2^ were necessary for this new finding, the results of this subsequent analysis are discussed in detail in a subsequent publication with common authors^1^. Though these new findings invalidate the conclusion in this Article on the arrangement of water molecules at the nanoparticle-water interface, they still demonstrate the high sensitivity of the developed subtraction method, namely the dd-PDF approach, in providing insight into the atomic and molecular arrangement of water and alcohol molecules in local ethanol-water motifs.

All authors agree to this retraction.
